# The effect of foot reflexology on fatigue in hemodialysis patients: a meta-analysis study

**DOI:** 10.1590/1518-8345.6804.4023

**Published:** 2023-10-06

**Authors:** Seda Şahan, Sevil Güler

**Affiliations:** 1 İzmir Bakircay University, Izmir, Menemen, Turkey.; 2 Erciyes University, Kayseri, Talas, Turkey.

**Keywords:** Hemodialysis, Fatigue, Reflexology, Foot Reflexology, Meta-Analysis, Hemodialysis Patients, Hemodiálisis, Fatiga, Reflexología, Reflexología Podal, Metanálisis, Pacientes de Hemodiálisis, Hemodiálise, Fadiga, Reflexologia, Reflexologia Podal, Metanálise, Pacientes de Hemodiálise

## Abstract

**Objective:**

this meta-analysis study analyzed the effect of foot reflexology on fatigue in hemodialysis patients by combining the results of independent studies on this subject.

**Method:**

meta-analysis study. A literature search was conducted in seven databases. The methodological quality of the included studies was assessed using tools proposed by the Joanna Briggs Institute. Comprehensive Meta-Analysis v3 was used for meta-analysis.

**Results:**

eight studies were included in the meta-analysis. The result of the meta-analysis standardized mean difference = 1.580 (95% Confidence Interval = 1.075 – 2.085 *p* = 0.000). The result of the subgroup analysis performed based on the number of foot reflexology sessions standardized mean difference = 1,478 (95% Confidence Interval = 1,210 – 1,747, *p* = 0.000).

**Conclusion:**

it was concluded that foot reflexology can be used to reduce fatigue in hemodialysis patients. No information was provided in the investigated studies about the possible side effects and negative effects of foot reflexology.

## Abstract (es)

**Objetivo:**

analizar el efecto de la reflexología podal sobre la fatiga en pacientes en hemodiálisis, combinando los resultados de estudios independientes sobre este tema.

**Método:**

estudio de metaanálisis. Se realizó una búsqueda bibliográfica en siete bases de datos. La calidad metodológica de los estudios incluidos se evaluó mediante las herramientas propuestas por el *Joanna Briggs Institute*. Para el metaanálisis se utilizó el programa *Comprehensive Meta-Analysis* v3.

**Resultados:**

en el metaanálisis se incluyeron ocho estudios. El resultado de la diferencia de medias estandarizada del metaanálisis = 1,580 (Intervalo de Confianza de 95% = 1,075 - 2,085 *p* = 0,000). El resultado del análisis de subgrupos realizado sobre la base de la diferencia de medias estandarizada en el número de sesiones de reflexología podal = 1,478 (Intervalo de Confianza de 95% = 1,210 - 1,747, *p* = 0,000).

**Conclusión:**

se concluyó que la reflexología podal puede utilizarse para reducir la fatiga en pacientes en hemodiálisis. En los estudios investigados no se proporcionó información sobre los posibles efectos secundarios y negativos de la reflexología podal.

## Abstract (pt)

**Objetivo:**

analisar o efeito da reflexologia podal sobre a fadiga em pacientes em hemodiálise, combinando os resultados de estudos independentes sobre este assunto.

**Método:**

estudo de metanálise. Foi realizada uma pesquisa bibliográfica em sete bases de dados. A qualidade metodológica dos estudos incluídos foi avaliada por meio de ferramentas propostas pelo *Joanna Briggs Institute*. Para a metanálise, foi utilizado o *Comprehensive Meta-Analysis* v3.

**Resultados:**

oito estudos foram incluídos na metanálise. O resultado da diferença média padronizada da metanálise = 1,580 (Intervalo de Confiança de 95% = 1,075 - 2,085 *p* = 0,000). O resultado da análise de subgrupo realizada com base na diferença média padronizada do número de sessões de reflexologia podal = 1,478 (Intervalo de Confiança de 95% = 1,210 - 1,747, *p* = 0,000).

**Conclusão:**

a reflexologia podal pode ser utilizada para reduzir a fadiga em pacientes em hemodiálise. Não foram fornecidas informações nos estudos investigados sobre os possíveis efeitos colaterais e negativos da reflexologia podal.

**Highlights:**

**(1)** This study found that foot reflexology reduced fatigue levels.

**(2)** The 10-session foot reflexology was the most effective.

**(3)** This study will guide nurses and health workers.

**(4)** Reflexology is one of the non-pharmacological methods used to relieve fatigue.

## Introduction

The application of hemodialysis, one of the treatment methods in individuals with chronic renal failure, aims to improve quality of life and prolong life span in patients^(^ 1 ^-^ 2 ^)^. The signs and symptoms of kidney failure are brought under control with the help of hemodialysis treatment. However, renal failure and hemodialysis treatment may cause other problems as well. Depending on the treatment, patients experience fluid and electrolyte imbalances, hypotension, muscle cramps, nausea, vomiting, fatigue, fever, uremic itching, tendency to infection, and endocrine anomalies. These effects cause physical, emotional, and psychological problems and deterioration of general health status^(^ 3 ^-^ 4 ^)^. Symptoms of fatigue, which occur as muscle weakness, waste accumulation and inflammatory processes and create a feeling of burnout in patients, are among the most critical problems associated with hemodialysis^(^ 5 ^)^. According to the literature^(6-7)^, fatigue was reported in 80-85% of the patient.

According to the literature, hemodialysis patients have lower physical functions compared to individuals with other chronic diseases^(^ 6 ^)^. It has been reported that patients with fatigue experience limitations in their social lives and physical activities, difficulties in business life, and difficulty making use of their spare time^(9-10)^.

Studies indicate that non-pharmacological methods should be used to manage symptoms such as weakness, muscle cramps, and fatigue caused by hemodialysis^(^ 7 ^-^ 8 ^)^. Yoga, meditation, acupressure, social support systems, and exercises are among the methods used to relieve the symptoms of fatigue^(^ 9 ^-^ 10 ^)^. Reflexology is one of the non-pharmacological methods used to relieve fatigue in hemodialysis patients^(^ 11 ^)^. Reflexology is a technique based on applying pressure to the reflex points on the feet, hands, and ears as representative of all organs, glands, and parts of the body^(^ 12 ^)^. Using pressure to the determined reflex points with the fingers helps maintain homeostasis and alleviates or even eliminates individuals’ health problems by non-pharmacological means^(^ 11 ^-^ 13 ^)^. Reflexology increases blood circulation, nerve stimulation, and endorphin release. Reflexology is a safe, easy-to-apply, cost-effective non-invasive practice that is effective for many symptoms when correctly applied^(^ 12 ^-^ 14 ^)^. It was reported^(^ 14 ^-^ 15 ^)^ that foot reflexology applied to hemodialysis patients decreased their fatigue levels and increased their physical activities.

This meta-analysis study analyzed the effect of foot reflexology on fatigue in hemodialysis patients by combining the results of independent studies on this subject.

## Method

This systematic review and meta-analysis was reported according to the updated Preferred Reporting Items for Systematic Reviews and Meta-Analyses (PRISMA) guidelines. The review protocol was registered in PROSPERO (CRD42020223148).

### Search strategy

A literature search was conducted in PubMed, Cochrane, Google Scholar, Scopus, ScienceDirect, Ovid Total Access, and EBSCO databases. Medical Subject Headings (MeSH) terms and text words were used as follows: (“hemodialysis” OR “dialysis”) AND (“foot reflexology” OR “reflexology”) AND (“fatigue’‘). The following filters were used in all databases in the search strategy:’‘Full text’‘,’‘2010-2022 years’‘,’‘Article types: randomized controlled trial, research articles’‘,’‘English Language’’. The studies that were accessed after the review were examined in regard to inclusion criteria to determine which studies to include in the meta-analysis. The literature review covered the studies conducted between 2010 and 2022. The literature review was carried out by two independent researchers.

### Inclusion criteria

The PICOS framework was used to determine the inclusion criteria for the meta-analysis: Population: adult patients (≥ 18 years old) undergoing hemodialysis therapy; Intervention: foot reflexology interventions; Comparison: patients in the control group received usual care. Patients in experimental group received foot reflexology. Pre- and post-test comparison in studies with a single sample group; Outcomes: studies that reported fatigue as a side effect of hemodialysis, and Study design: randomized controlled studies, case-control studies, experimental studies, quasi-experimental studies. Others inclusion criteria: studies conducted between 2010 and 2022; that provided the necessary findings for meta-analysis; studies with full text available and published in English language.

### Exclusion criteria

The study excluded the following: reviews, systematic reviews and meta-analyses, case reports, descriptive studies and articles that were not suitable for the purpose of the study.

### Data selection and extraction

Results from electronic databases were downloaded and saved in EndNoteX9. Duplications were identified and removed through the EndNoteX9 program. After subtracting duplications, the remaining results were scanned by two investigators (SŞ and SG) - titles and abstracts. The researchers then reviewed the studies’ full texts that met the inclusion criteria. After taking the full text of potentially relevant articles and uploading them to the EndNote software, the researchers conducted a full-text-level evaluation to select the appropriate articles to be included in this meta-analysis. Finally, studies that met the inclusion criteria in the meta-analysis were identified. At each step, disagreements and inconsistencies were discussed and resolved with an independent reviewer until a consensus was reached.

A data coding system was used for the included articles in the final stage. Author, year, origin of the studies, study design, intervention time and sessions, scales, analyses used, pre and post-test results, and test results such as t and *p* were recorded. Each researcher separately extracted the characteristics of the studies. It has been checked and verified on two datasets by an independent researcher. Any disagreements were resolved through discussion among all authors. The authors were contacted for further information if necessary (for example, if data were not explicitly reported).

### Literature quality appraisal (Risk of Bias in Included Studies)

The Joanna Briggs Institute Checklist (JBI) was used for quasi-experimental studies and randomized controlled trials. According to JBI, quality assessment of randomized controlled trials are evaluated in 13 categories. Quasi-experimental studies are evaluated in nine categories. The risk of bias was rated as unclear, low, and high. The risk of bias for each trial was assessed independently by five review. Two of the evaluators are the authors of this study and three are independent evaluators from the study. Each study was reviewed to determine whether it had minimized the possibility of bias in its design, conduct, and analysis. Each study was scored based on adherence to the appraisal tools (Yes = 1, Unclear or No = 0), and an overall percentage was applied^(^ 16 ^)^. According to the results of the quality evaluation made according to JBI, score and risk status of the studies are given in Figure 2.

It was summarized using The Grading of Recommendations, Assessment, Development and Evaluation (GRADE) approach, which assesses evidence from multiple perspectives, including certainty, study design, risk of bias, inconsistency, indirectness, and precision. GRADE divides the certainty of evidence into four levels: high, moderate, low, and very low.

The assessment was conducted in GRADEpro by the primary author and verified by a second author.

### Data analysis method

Comprehensive Meta Analysis V3 (CMA), a statistical package program, was used for meta-analysis. EndNote X9 program was used to store the studies obtained after review to separate duplicates and Microsoft Office Excel program was used to save the data of these studies and transfer them to CMA.

As the studies used different measures for the outcomes of interest, the standardized mean difference was calculated (standardized mean difference) to determine the effects of the intervention in the experimental group compared to the control group. Effect size magnitudes were interpreted as 0.2 ≤ standardized mean difference < 0.5 = small, 0.5 ≤ standardized mean difference < 0.8 = moderate, and 0.8 ≤ standardized mean difference = large. When the studies measured outcomes at multiple follow-up time points, post-test outcome measures, conducted immediately after the intervention, were used.

This study utilized the standardized effect size when the effect sizes were calculated for each study^(^ 17 ^)^. The standardized mean difference was calculated at the 95% confidence interval (lower and upper limit). I^2^, *p* and Q values were evaluated to determine the level of heterogeneity. An I^2^ value higher than 75% indicates marked heterogeneity between studies and 75%, 50%, 25%, and 0% were shown as high, moderate, low, and no heterogeneity, respectively^(^ 18 ^)^.

The meta-analysis results pointed to high heterogeneity (I^2^=86,670). For this reason, the random effects model was used in the interpretation of the meta-analysis results. In addition, subgroup analyzes were performed for factors such as the scale used in the studies, the duration of hemodialysis, and the foot reflexology practice session.

## Results

**Figure 1 -:**
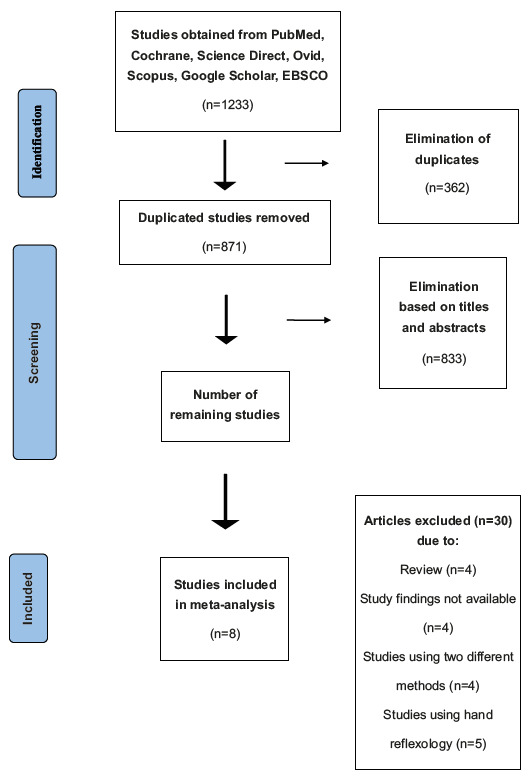
PRISMA Flowchart of study selection. İzmir, Turkey, 2019-2020

A total of 1233 studies were reached as a result of the literature review. Following the PRISMA Flowchart (Figure 1), a total of eight studies^(^ 14 ^-^ 15 ^,^ 19 ^-^ 20 ^)^ were included in the meta-analysis (Figure 1).

Two of these studies^(^ 14 ^,^ 21 ^)^ were randomized controlled and six studies^(^ 22 ^-^ 15 ^,^ 18 ^-^ 23 ^)^ were quasi-experimental. The total sample size of the studies included in the meta-analysis was 216 for the control group and 273 for the experimental group. The studies^(^ 14 ^-^ 15 ^,^ 18 ^-^ 21 ^)^ were conducted with a small sample group (n<50). The characteristics of the studies included in the meta-analysis are given in Figure 2.

**Figure 2 -:** Characteristics of studies included in meta-analysis (n = 8). İzmir, Turkey, 2019-2020

**Author (Year)** **Country**	**Design**	**Sample size**	**Scale**	**Intervention**	**Hemodialysis frequency (times** per **week)**	**Frequency and duration**	**Risk of Bias Assessments**
Özdemir, et al. (2013) Turkey^(^ 14 ^)^	Randomized Controlled Research	Experimental Group: 40 Control Group: 40	Piper Fatigue Scale	Experimental Group: Usual care + Foot Reflexology Control Group: Usual Care	3	3 times/week 30 minutes/time Total weeks: 1 Total session:3	Low Risk (10 points)
Ünal and Akpınar (2016) Turkey^(^ 20 ^)^	Randomized Controlled Research	Experimental Group:36 Control Group: 37	Visual Analog Fatigue Scale	Experimental Group: Usual care + Foot Reflexology Control Group: Usual Care	2	2 times/week 30 minutes/time Total weeks:4 Total session:8	Low Risk (10 points)
Anushamole, et al. (2016) Indıa^(^ 15 ^)^	Quasi-experimental Research	Experimental Group:30 (Pre-post test)	Piper Fatigue Scale	Experimental Group: Usual care + Foot Reflexology	2	2 times/week 30 minutes/time Total weeks:3 Total session:6	Low Risk (8 Points)
Bazzi, et al. (2017) Iranian^(^ 24 ^)^	Quasi-experimental Research	Experimental Group: 26 Control Group: 26	Fatigue Severity Scale	Experimental Group: Usual care + Foot Reflexology Control Group: Usual Care	3	2 times/week 30 minutes/time Total weeks:5 Total session:10	Low Risk (9 points)
Sharifi, et al. (2018) Iran^(^ 21 ^)^	Quasi-experimental Research	Experimental Group: 45 Control Group: 43	Fatigue Severity Scale	Experimental Group:Usual care + Foot Reflexology Control Group: Usual Care	3	3 times/week 30 minutes/time Total weeks:1 Total session:3	Low Risk (9 points)
Ahmadidarrehsima, et al. (2018). Iran^(^ 19 ^)^	Quasi-experimental Research	Experimental Group: 26 (Pre-post test)	Fatigue Severity Scale	Experimental Group:Usual care + Foot Reflexology	3	2 times/weeks 30 minutes/time Total weeks:3 Total session:6	Low Risk (8 Points)
Shady and Ali (2019) Egypt^(^ 22 ^)^	Quasi-experimental Research	Experimental Group: 36 Control Group: 36	Multidimensional Assessment of Fatigue Scale	Experimental Group: Usual care + Reflexology Control Group: Usual Care	3	3 times/week 30 minutes/time Total weeks:3 Total session:9	Low Risk (9 points)
Jumadi, et al. (2019) Indonesia^(^ 23 ^)^	Quasi-experimental Research	Experimental Group: 34 Control Group: 34	Multidimensional Assessment of Fatigue	Experimental Group: Usual care + Foot Reflexology Control Group: Usual Care	3	3 times/week 30 minutes/time Total weeks: 1 Total session: 3	Low Risk (9 points)

**Figure 3 -:**
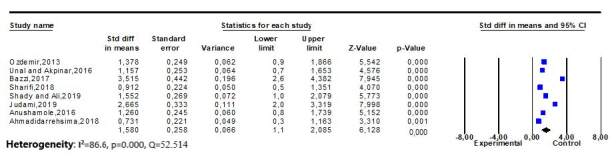
Effect size of studies and forest plots (n = 8). İzmir, Turkey, 2019-2020

Heterogeneity test was performed by calculating the statistical value of Q (52.514), *p* (0.000) and I^2^ (86.67). These values indicated that the study was highly heterogeneous. For this reason, random effects model was used to calculate the overall effect size in the study. Figure 3 presents the findings regarding the heterogeneity test and the overall effect size.

According to the result of the meta-analysis, the mean effect size was determined to 1.580 (95% CI = 1.075 – 2.085; *p*<0.001), i.e., very strong, based on the random-effects model. According to this result, foot reflexology reduced fatigue levels in hemodialysis patients. The effect size of all studies were found to be positive (intervention was in favor of the experimental group) (Figure 3).

Due to the high heterogeneity of the meta-analysis, subgroup analyses were conducted to determine the source of heterogeneity and the factors affecting the meta-analysis result. First of all, the scale types used to determine the effect of foot reflexology on fatigue were examined in the investigated studies. A significant difference was found between the scales used to determine the effect of foot reflexology on fatigue according to the results of the subgroup analysis conducted according to the scale types used in the studies which were included in the meta-analysis (*p*=0.004). The result of the meta-analysis based on the scales showed a very strong mean effect size (Q_between_) which was 1.503 (95% CI = 1.275 – 1.735; p = 0.004).

There was a significant difference between the scales and by looking at the effect sizes, we can argue that The Multidimensional Assessment of Fatigue, Fatigue Severity Scale, Multidimensional Fatigue Scale, Visual Analog Fatigue Scale and Piper Fatigue Scale can be used respectively in regards to the effect of foot reflexology on fatigue (Table 1).

**Table 1 -:** Subgroup analysis according to scales used in the studies (n = 8). İzmir, Turkey, 2019-2020

Scales	Study Number	Standard Mean Effect	Standard Error	95% IC*		Z^†^	p^‡^	
				Lower Limit	Upper Limit			
MAF^§^	1	2,665	0,333	2,012	3,319	7,998	0,000	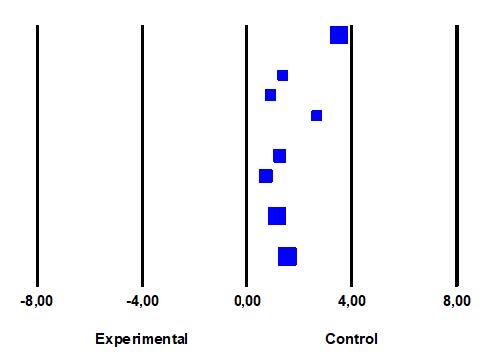
FSS ^||^	3	1,652	0,646	0,386	2,918	2,557	0,011	
MFS ^¶^	1	1,552	0,269	1,025	2,079	5,773	0,000	
VAFS**	1	1,157	0,253	0,662	1,653	4,576	0,000	
PFS^††^	2	1,318	0,174	0,976	1,660	7,559	0,000	
Q_between_^‡‡^	8	1,503	0,116	1,275	1,731	12,910	0,004	

*CI = Confidence Interval; ^†^Z = Effect Size; ^‡^p = p<0.05; ^§^MAF = Multidimensional Assessment of Fatigue; ^||^FSS = Fatigue Severity Scale; ^¶^MFS = Multidimensional Assessment of Fatigue Scale; ^**^VAFS = Visual Analog Fatigue Scale; ^††^PFS = Piper Fatigue Scale; ^‡‡^Q_between_ = Total mean size between scales

Subgroup analyzes were conducted based on the number of sessions to determine the effect of the foot reflexology application sessions on fatigue. There was a significant difference between the number of sessions applied to determine the effect of foot reflexology on fatigue based on the results of the subgroup analysis performed according to the foot reflexology sessions in the studies in the meta-analysis (*p*<0.001). The average effect size (Q_between_) was very strong with standardized mean difference = 1.478 (95% CI = 1.210 – 1.747; p = 0.000) as a result of the subgroup meta-analysis based on the number of sessions.

However, since there is a significant difference between the number of sessions according to the Q_between_ value, we can argue that the most effective application in the effect of foot reflexology on fatigue was achieved by performing 10 sessions, followed by three sessions, nine sessions, eight sessions and six sessions, respectively (Table 2).

**Table 2 -:** Subgroup analysis of foot reflexology by practice session (n = 8). İzmir, Turkey, 2019-2020

Session	Study Number	Standard Mean Effect	Standard Error	95% IC*		Z^†^	p^‡^	
				Lower Limit	Upper Limit			
10 session	1	3,515	0,442	2,648	4,382	7,945	0,000	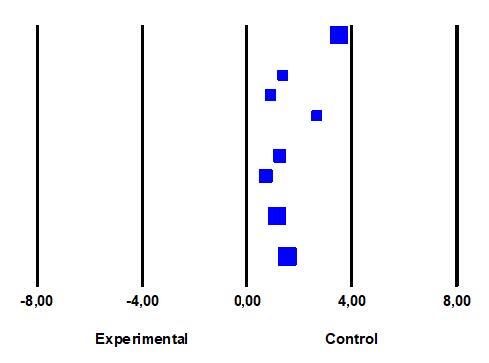
3 session	3	1,624	0,473	0,697	2,550	3,434	0,001	
9 session	1	1,552	0,269	1,025	2,079	5,773	0,000	
8 session	1	1,157	0,253	0,662	1,653	4,576	0,000	
6 session	2	0,985	0,264	0,497	1,503	3,724	0,000	
Q_between_^§^	8	1,478	0,137	1,210	1,747	10,798	0,000	

*CI = Confidence Interval; ^†^Z = Effect Size; ^‡^p = p<0.05; ^§^Q_Between_ = Total mean size between scales

The heterogeneity could derive from various sources, e.g., participant’s demographic information, sample size, studies design, outcome measure, duration of follow-up, tc. In order to investigate heterogeneity, sensitivity analyzes were conducted and the heterogeneity was constantly existence. In another sensitivity analysis of only studies that lower standard mean effect, the pooled adjusted from seven studies was 1.580 (95% CI = 1.075 – 2.085, *p*<0.001). Additional sensitivity analyses excluding one study at a time and then pooling the remaining studies showed high heterogeneity (I^2^=86.67) and standardized mean difference 1.580 (95% CI = 1.075 – 2.085, *p*<0.001). Therefore, the random effects model was adopted in the final meta-analysis process to incorporate in the heterogeneity among the studies.

Classic fail-safe N test was used to determine publication bias. For an alpha value of 0.05, the number of studies was found to be 505 based on the relevant calculation. Insignificance of the *p*-value-2-tailed value in the Begg and Mazumdar Rank Correlation analysis, another indicator of publication bias, points to no publication bias (*p*-value-2-tailed = 0.29902 >0.05). Another bias test is Duval and Tweedie’s trim-and-fill analysis^(^ 25 ^)^. According to the trim-and-fill analysis, there was no study to be added to the left of the funnel diagram, but if one study is added to the right, the filled effect size would be the same as the effect size found in this study. These results also indicated that there was no significant publication bias.

## Discussion

This meta-analysis study analyzed the effect of foot reflexology on fatigue in hemodialysis patients by combining the results of independent studies on this subject and presented a meta-analysis of the research findings.

Fatigue is one of the most common adverse effects of chronic renal failure and hemodialysis. Hemodialysis-related fatigue adversely affects the quality of life in patients^(5,12,30)^. The literature reports that hemodialysis patients have moderate to high fatigue statuses^(^ 24 ^,^ 21 ^-^ 20 ^,^ 26 ^)^.

Complementary and alternative medicine offers frequently used applications in health care services. Generally, patients turn to complementary and alternative treatment practices because they fear the side effects of drugs. Nurses are getting to be more involved in these practices^(^ 27 ^-^ 28 ^)^. Therefore, reducing the level of fatigue and making interventions in this regard are important responsibilities of nurses.

One of the oldest treatments in the world, reflexology is one of the complementary and alternative treatment methods and it is based on the scientific massage technique developed in China since the ancient times^(^ 29 ^)^. In reflexology, the pressure applied to the reflex points of the body in the ears, hands, and feet provides a healing effect on the body systems by stimulating the inactivated areas in the body and soothing the over-activated areas^(^ 30 ^)^.

The result of the meta-analysis showed the mean effect size to be very strong and 1.580 (95% CI = 1.075 – 2.085, *p*<0.001) based on the random effects model. According to this result, foot reflexology reduced fatigue in hemodialysis patients. According to the another meta-analysis^(^ 31 ^)^, it was found that foot reflexology reduced fatigue. However, different patient groups were included in the study. Specifically, the findings for hemodialysis patients were not presented.

The total sample size of the studies^(^ 22 ^-^ 15 ^,^ 19 ^-^ 21 ^)^ included in the meta-analysis was 216 for the control group and 273 for the experimental group. It was found that the studies were carried out with a small sample group (n<50). The number of samples is essential to generalize a research result and make high-powered decisions regarding the results of prospective meta-analysis studies. As the number of samples increases, the meta-analysis effect size increases as well^(^ 32 ^)^. It can be argued that the study group consisted of patients receiving continuous hemodialysis, affecting the sample size due to refusal or withdrawal from the treatment, so it mainly was studied with a smaller sample group. In addition, the studies did not provide adequate information about the possible side effects and adverse effects of foot reflexology on patients. Therefore, it is essential to consider these aspects in future studies.

Multidimensional Assessment of Fatigue (MAF) scale was the first in the list when the order of the effect level was considered according to the mean effect sizes of the scales. In this case, we can say that the MAF scale addresses fatigue with various dimensions.

It can be recommended to apply 10 foot reflexology sessions primarily in hemodialysis patients based on the mean effect sizes of the applied reflexology sessions. However, studies showed that foot reflexology practice sessions were significant for each session (p<0.05). For this reason, patients who cannot or do not want to have a long-term application, three sessions of application, which are listed as second according to the average effect size, can be used in cases where the effect wants to be obtained in a short time.

All the studies in the meta-analysis reported a total of 30 minutes of application, 15 minutes on each foot. Since foot reflexology was applied simultaneously in the studies, subgroup analyses could not be performed. However, in two studies^(^ 31 ^,^ 33 ^)^ with people with premenstrual syndrome (PMS), 60 minutes was the standard time for reflexology with the most significant effect on the total PMS score. No information was given regarding the duration of foot reflexology application in the studies. Since the meta-analysis results showed that foot reflexology was effective on fatigue in hemodialysis patients, 30 minutes of application time can be sufficient. However, using different application times may change the effect of foot reflexology on fatigue. For this reason, it may be recommended to compare different application periods in future studies.

## Conclusion

This study found that foot reflexology reduced fatigue levels in hemodialysis patients. The order of the effect level according to the mean effect sizes of the reflexology sessions showed that the 10-session foot reflexology was the most effective. These results contain important findings for controlling and reducing fatigue in clinical practice. The most effective number of sessions and evaluation scales are presented for nurses who will practice foot reflexology. Therefore, we can argue that the meta-analysis results obtained in this study will guide the nurses. At the same time, nurses should be offered training and practice on this subject since medical treatment for fatigue in hemodialysis patients can be reduced with foot reflexology.

Blinding and randomization were not specified in the two randomized controlled trials included in the meta-analysis. This situation reduces the quality appraisal of the studies and affects the reliability of the study results. For this reason, it is recommended that the cases of randomization and blinding should be clearly stated in future studies.

No information was provided in the investigated studies about the possible side effects and negative effects of foot reflexology. According to the results of this study, we can argue that foot reflexology positively affects fatigue, but we cannot present any information about the side effects that may be experienced by patients. Providing detailed information about the negative effects in future studies will provide more detailed meta-analysis results.
